# The Mexican Version of the Interactive mHealth App Usability Questionnaire (Mx-MAUQ) in Women With Breast Cancer: Instrument Validation Study

**DOI:** 10.2196/72215

**Published:** 2025-08-29

**Authors:** Saúl Eduardo Contreras-Sánchez, Svetlana V Doubova, Iris Romero-Espinal, María Fernanda González-Garnica, Hannah H Leslie

**Affiliations:** 1 Epidemiology and Health Services Research Unit CMN Siglo XXI, Mexican Institute of Social Security, Av. Cuauhtémoc 330, Col. Doctores, Mexico City, 6720, Mexico, 52 5556276900 ext 21072; 2 Division of Prevention Science, University of California San Francisco, San Francisco, CA, United States

**Keywords:** psychometric validation, responsive web application, usability, Mexico, mHealth

## Abstract

**Background:**

Successful eHealth applications require careful assessment to ensure their ease of use, usefulness, and user satisfaction. Responsive web applications are eHealth tools that operate on any internet-enabled device across all browsers. Psychometrically valid assessment tools are essential for effectively evaluating these applications, yet no validated eHealth questionnaire exists for assessing their usability and user satisfaction in Mexico.

**Objective:**

The objective of this study is to adapt the mHealth App Usability Questionnaire (MAUQ) for responsive web application assessment in Mexico and validate adapted Mx-MAUQ content, construct validity, internal consistency, and its ability to distinguish between patient subgroups.

**Methods:**

We conducted a psychometric validation study of Mx-MAUQ with women aged 20 to 75 diagnosed with stage I-III breast cancer who had begun neoadjuvant or adjuvant treatment within the last six weeks and used the responsive “OncoMama App” for 1 month. The study excluded women with stage IV breast cancer, illiterate women, and those with blindness, cognitive disability, or severe depression. Participants were recruited from oncology services at 4 hospitals belonging to the Mexican Institute of Social Security between August 2023 and November 2024. The study involved translating and adapting the MAUQ while evaluating its content through expert panels and cognitive interviews with women. The Mx-MAUQ construct was assessed through exploratory factor analysis (EFA), internal consistency via Cronbach α, and Mx-MAUQ’s capacity to distinguish between subgroups of patients with breast cancer using the Wilcoxon rank sum test.

**Results:**

A total of 210 women participated, with 75.2% (n=158) aged 60 or younger and 64.3% (n=135) having high school education. The expert panel granted all Mx-MAUQ items a content validity index (CVI) above 0.7. Experts have found that the MAUQ questions are general enough to be relevant not only to mobile apps or specific medical conditions but also to a variety of digital platforms, including responsive web applications and different health conditions. The cognitive interviews revealed 3 unclear terms in the questionnaire; consequently, we defined “application interface” and changed “social settings” to “social environments” and “manage my health” to “take care of my health.” EFA identified 2 factors explaining 91.6% of the variance and retaining all items. The first factor, “Ease of Use,” consists of 9 items and has a Cronbach α of .94. The second factor, “Satisfaction, Usefulness, and System Information Arrangement,” includes 12 items and has a Cronbach α of .97. Women with higher education levels scored significantly higher for both factors, as well as the overall Mx-MAUQ score, than those with lower educational attainment.

**Conclusions:**

Mx-MAUQ showed satisfactory psychometric properties based on EFA, internal consistency, and discriminant analysis, making it a suitable tool for a comprehensive assessment of the usability of interactive web-based eHealth applications for women with breast cancer in Mexico.

## Introduction

Over the past three decades, the integration of digital technologies in health services has offered significant opportunities to address health care systems challenges, enhancing access to and quality of care through person-centered digital interventions [1].

The successful integration of digital or eHealth interventions in health practice depends on their rigorous development, implementation, and assessment [2]. These processes should guarantee that a digital system allows users to perform tasks easily, safely, and effectively, while getting a satisfactory experience [3]. Consequently, having psychometrically valid tools to assess the usability of digital health applications is an essential requirement.

In Mexico, the federal government acknowledges the importance of eHealth strategies to improve access, coverage, and quality of health services nationwide, yet research and implementation of eHealth in the country is limited.

Breast cancer is the leading cause of morbidity and mortality from malignant diseases among Mexican women [4]. Many women in public hospitals report unmet supportive care needs and care quality issues [5,6]. To address these challenges, we developed an eHealth intervention featuring a responsive web application (named “OncoMama App”) for tracking symptoms and supportive care needs, along with proactive follow-up by nurses [7]. Responsive web applications are web-based eHealth tools that work on any device with Internet access and can be opened in any browser. Users can access them via a URL, which eliminates the need for app store downloads. Additionally, they are generally cheaper to develop and easier to update than mobile apps [8]. The “OncoMama App” is an interactive responsive web application for patients with breast cancer. It captures personal and clinical data, tracks symptoms, and supportive care needs. The application includes tools to assess quality of life, links to resources from breast cancer organizations in Mexico, and the ability to automatically send emails with non-pharmacological recommendations for patients with breast cancer and with mild symptoms. For individuals experiencing and registering moderate to severe symptoms, instant automated notifications are sent to nurses to follow them up via phone [7,9].

The comprehensive evaluation of web-based eHealth applications requires appropriate tools. In Mexico, the System Usability Scale (SUS) was previously adapted and validated [10,11]. This questionnaire aims to assess the usability of general electronic systems, with a primary focus on ease of use; however, it overlooks elements such as system usefulness, system information arrangement, and user satisfaction. Additionally, the Computer Systems Usability Questionnaire (CSUQ) has been validated [12]. However, this questionnaire also lacks questions related to usefulness. Furthermore, neither of these questionnaires was developed specifically for eHealth applications.

The mHealth App Usability Questionnaire (MAUQ) is a comprehensive, psychometrically validated tool designed to assess the usability of mobile health (mHealth) apps [13]. Developed in English in the United States, MAUQ has 4 versions; each version is tailored to the type of app interaction—standalone or interactive—and the user—patients or providers. MAUQ measures ease of use and satisfaction, system information arrangement, and usefulness of mobile apps. The patient versions of the MAUQ have been translated and validated for various countries, populations, and types of mHealth apps, including the Enable app for patients with cancer in Germany [14], the DIGICOG-MS app for individuals with multiple sclerosis in Italy [15], the LactApp for breastfeeding women in Spain [16], the “Good Doctor” and “Left-handed Doctor” mHealth apps in China [17,18], and the MyFitnessPal app in Malaysia [19]. Nonetheless, it has not yet been validated in Mexico, even its comprehensive nature and emphasis on eHealth, unlike the SUS and CSUQ, which do not possess these features. Therefore, the objective of this study was to translate the interactive version of the MAUQ for patients into Mexican Spanish, adapt it for the responsive web application, and perform psychometric validation of the translated and adapted questionnaire.

## Methods

We conducted the psychometric validation study of MAUQ as part of a broader research project designing and evaluating an eHealth intervention that combines a responsive web application registry to capture symptoms and supportive care needs of women with breast cancer and their proactive follow-up by nurses. The design, implementation, and evaluation protocol of this intervention have been published elsewhere [7].

### Study Population and Eligibility Criteria

The study population included women between 20 and 75 years diagnosed with stage I-III breast cancer who have started neoadjuvant or adjuvant treatment with chemotherapy or radiotherapy within the past 6 weeks; have access to the internet via mobile phone, computer, or tablet; and provided written informed consent. The study excluded women with stage IV breast cancer, illiterate women, and those with blindness or low vision not corrected with glasses, cognitive disability (eg, Alzheimer disease, other dementias, or other mental illness with intellectual disability), or severe depression (≥12 points on the Hospital Anxiety and Depression Scale).

Participants received training from the study nurse on using the “OncoMama App” in their cellphones before its use. They were advised to use it for registering cancer symptoms or adverse treatment effects or to record the absence of symptoms weekly.

### Study Location

Participants were recruited from oncology services at 4 hospitals belonging to the Mexican Institute of Social Security (IMSS) (three in Mexico City and one in Puebla) between August 2023 and November 2024. IMSS is the largest public health service in Mexico, delivering health care to more than 70 million individuals (57% of the population), primarily workers in the formal labor market and their families. The hospitals chosen for the study were selected based on convenience, encompassing all facilities in Mexico City and Puebla that provide care for women with breast cancer.

### Study Variables

The primary study variable was the usability of a responsive web application. To measure this variable, we performed translation, adaptation, and psychometric validation of the interactive version of MAUQ for patients. This questionnaire measures the ease of use, satisfaction, system information arrangement, and usefulness of the digital app. It has 21 items measured on a 7-point Likert-type scale (from strongly disagree to strongly agree) and grouped in three domains: (1) ease of use and satisfaction (8 items); (2) system information arrangement (6 items); and (3) usefulness (7 items). The overall score is obtained by adding the scores of all items and dividing it by the total number of items.

Information was also collected on the demographic and clinical characteristics of patients. These variables included age (<60 y or ≥60 y), education level (secondary school or lower, or high school and above), cancer stage (I, II, or III), type of treatment (radiation therapy or chemotherapy), and the presence of chronic diseases prior to the cancer diagnosis (yes or no).

### MAUQ Validation Process

The validation of the MX-MAUQ was conducted in two stages: (1) translating the MAUQ and adapting and evaluating content validity, considering the web-based nature of the application, and (2) assessing its construct validity, internal consistency, and subgroup differentiation abilities.

#### Stage 1

The MAUQ translation followed the recommendations of the World Health Organization to ensure quality and consistency with the original questionnaire [20]. A bilingual translator translated it into Mexican Spanish, and another translator performed a back-translation into English. Following this, a review was conducted to verify the consistency of the back-translated version with the original questionnaire, leading to the creation of the final translation (Multimedia Appendix 1). No significant changes were made to the wording of the original MAUQ during the translation process. A panel of experts comprising 3 oncologists, 1 radiation oncologist, 2 oncology nurses, a psychologist, and a health services researcher conducted the content validation of the Mx-MAUQ. Due to the “OncoMama App” focusing on patients with breast cancer, the expert group consisted of individuals who provide care for these patients at IMSS and have experience using mobile and web applications and questionnaire validation. The number of experts was based on Lynn’s suggestion, recommending at least 5 and no more than 10 to ensure adequate control of random agreement [21]. Prior to the validation of the MAUQ content, the expert group had access to the “OncoMama App” and engaged with it for 3 weeks to become acquainted with its features. Following this interaction with the application, the experts assessed the relevance of each Mx-MAUQ item to evaluate the usability of the studied responsive web application and the appropriateness of its language. Each item was rated as follows: 1=“not relevant,” 2=“useful but not relevant or not feasible,” or 3=“relevant” to assess the usability of the responsive web application. The experts’ responses were compiled, and the content validity index (CVI) was calculated using the formula (ne-N/2)/(N/2), where “ne” is the number of experts who deemed the item relevant, and “N” is the total number of experts [22].

After the expert group’s validation of the questionnaire, we conducted 2 rounds of cognitive interviews [23], each with 9 patients with breast cancer, to assess the clarity, comprehension, and relevance of the content of Mx-MAUQ perceived by women. To achieve saturation, we followed Willis et al.’s recommendation of conducting 1-3 rounds of 8-12 interviews each [24]. The expert group reviewed the interviews, identifying and modifying terms that were misunderstood or irrelevant to the women interviewed.

#### Stage 2

Construct validity was conducted by exploratory factor analysis (EFA). The sample size for construct validity was estimated at around 10 patients per question of the questionnaire to obtain adequate stability of the factorial structure during the validation process [25], with a minimum required sample of 210 patients. For this analysis, we used responses from patients with breast cancer participating in the eHealth intervention with a web application registry, who completed the Mx-MAUQ during the first assessment after 1 month of application use. The questionnaire was self-administered, and the data were collected through a web application.

The exploratory factor analysis used the principal axis factorization method based on a polychoric correlation matrix [26], due to the asymmetric distribution of most items of the questionnaire. The Promax oblique rotation was used based on the assumption that the factors are related to each other. The relevance of this analysis was confirmed by the Kaiser-Meyer-Olkin (KMO) sampling adequacy measure greater than 0.5 and Bartlett’s sphericity test, which proved that the observed correlation matrix was different from the identity matrix [27]. The number of factors was determined by (1) the number of eigenvalues greater than 1.0 and (2) the sedimentation graph, considering the cut-off point where the graph curve becomes horizontal. Factor loadings ≥0.4 were considered significant, so items with lower factor loadings and those items associated with more than 1 factor should be eliminated from the analysis [28]. In addition, the percentage of variance explained by each factor and the communality of each item were calculated.

The questionnaire’s reliability was assessed using Cronbach α, which helped evaluate the internal consistency of items within the same category, with values greater than 0.7 deemed acceptable [29].

The Mx-MAUQ’s ability to differentiate between subgroups of women with breast cancer was evaluated through the Wilcoxon rank sum test, which compared the median Mx-MAUQ scores (by factor and total score) of women from different subgroups considering characteristics such as age, educational attainment, chronic comorbidity, cancer stage, and treatment type. We hypothesized that patients aged 60 and older, with secondary education or less, chronic diseases, advanced cancer (stage III), and those undergoing radiation therapy would report lower usability of responsive web applications compared to their counterparts.

In addition, a descriptive analysis was performed to describe the women’s demographic and clinical characteristics, as well as to examine the distribution of responses to each item of the Mx-MAUQ using frequencies and percentages of the categorical variables and median with interquartile range of the numerical variables, due to their non-normal distribution.

Stata V.17.0 (Stata) was used for the analysis and a *P* value of≤.05 was considered statistically significant.

### Ethical Considerations

The study was authorized by the National Research and Ethics Committees of the Mexican Institute of Social Security (R-2021-785-059). It was conducted in accordance with the ethical principles outlined in the Declaration of Helsinki. Participants signed an informed consent form prior to joining the study. The researchers ensured the privacy and confidentiality of the participants' data and identities. All electronic data were stored on password-protected computers, and only deidentified data are reported in this paper and the supplementary material. Participants in the study did not receive any compensation for their involvement.

## Results

### Content Validation and Cognitive Interviews

During content validation, all items received a CVI above 0.7 from the expert panel (see Multimedia Appendix 2), eliminating the need for any removals. However, some items were reordered within domains for better flow of questions. Furthermore, the first round of cognitive interviews identified several unclear terms, including “application interface,” “social settings,” and “manage my health.” To make these terms clear, we incorporated the definition of the term “application interface,” describing it as “the visual way in which the contents and images of the application are presented” (item 2). Similarly, we modified the terms “social settings” to “social environments” (item 5) and “manage my health” to “take care of my health” (item 17). The second round of interviews confirmed clarity and understanding of the revised items.

### Descriptive Analysis

Table 1 presents the demographic and clinical characteristics of 210 patients with breast cancer who were included in the construct validation phase of the questionnaire and completed the 1-month evaluation of the “OncoMama App.” These patients included 50 women from the pilot test of the clinical trial and 160 women from the actual clinical trial. The data reflects the responses of those who completed the 1-month assessment first. The median age of the women was 51 years, with 75.2% (n=158) being 60 or younger. Over half had a high school education or higher (n=135, 64.3%), and about one-third had a chronic condition before cancer diagnosis; 42.4% (n=89) were at clinical stage III, and 88.1% (n=185) received chemotherapy.

**Table 1.** Women demographic and clinical characteristics (N=210).

Women characteristics	Values
Age (years), median (quartile 1, 3)	51 (42-59)
Age group, n (%)	
<60 years	158 (75.2)
≥60 years	52 (24.8)
Educational attainment, n (%)	
Secondary school or lower	75 (35.7)
High school or higher	135 (64.3)
Cancer stage, n (%)	
I-II	121 (57.6)
III	89 (42.4)
Treatment type, n (%)	
Radiation therapy	25 (11.9)
Chemotherapy	185 (88.1)
Chronic comorbidity, n (%)	
No	141 (67.1)
Yes	69 (32.9)

There were no missing responses on Mx-MAUQ questionnaire items. Respondents tended to agree or strongly agree with the items in the Mx-MAUQ instrument. The most endorsed items were: “I felt comfortable communicating with my health care provider using the app” (n=124, 59.1% strongly agree and n=59, 28.1% agree); “The app made it convenient for me to communicate with my health care provider” ( n=119, 56.7% and n=67, 31.9%); “The app would be useful for my health and well-being” (n=116, 55.2% and n=71, 33.8%); and “Overall, I am satisfied with this app” (n=115, 54.8% and n=67, 31.9%). The items that showed the lowest percentage of “strongly agree” responses were “Whenever I made a mistake using the app, I could recover easily and quickly” (n=77, 36.7%) and “This app has all the functions and capabilities I expect it to have” (n=83, 39.5%) (as shown in Table 2).

**Table 2.** Descriptive statistics of the Mexican version of the interactive mHealth App Usability Questionnaire (Mx-MAUQ) (N=210).

Items	Strongly disagree, n (%)	Disagree, n (%)	Some-what disagree, n (%)	Neither agree nor disagree, n (%)	Some-what agree, n (%)	Agree, n (%)	Strongly agree, n (%)
Q1: It was easy for me to learn to use the app.	3 (1.4)	9 (4.3)	16 (7.6)	22 (10.5)	18 (8.6)	51 (24.3)	91 (43.3)
Q2: I like the interface of the app.	3 (1.4)	1 (0.5)	2 (0.9)	13 (6.2)	14 (6.7)	79 (37.6)	98 (46.7)
Q3: The app was easy to use.	4 (1.9)	4 (1.9)	15 (7.1)	25 (11.9)	16 (7.6)	57 (27.1)	89 (42.4)
Q4: The information in the app was well organized, so I could easily find the information I needed.	2 (0.9)	3 (1.4)	9 (4.3)	15 (7.1)	17 (8.1)	66 (31.4)	98 (46.7)
Q5: I feel comfortable using this app in social settings.	2 (0.9)	2 (0.9)	5 (2.4)	19 (9.1)	22 (10.5)	60 (28.6)	100 (47.6)
Q6: The amount of time involved in using this app has been fitting for me.	3 (1.4)	4 (1.9)	7 (3.3)	17 (8.1)	17 (8.1)	67 (31.9)	95 (45.2)
Q7: I would use this app again.	1 (0.5)	1 (0.5)	2 (0.9)	14 (6.7)	15 (7.1)	63 (30.0)	114 (54.3)
Q8: Overall, I am satisfied with this app.	1 (0.5)	2 (0.9)	1 (0.5)	10 (4.8)	14 (6.7)	67 (31.9)	115 (54.8)
Q9: This mHealth app provided an acceptable way to receive health care services.	1 (0.5)	1 (0.5)	2 (0.9)	14 (6.7)	9 (4.3)	70 (33.3)	113 (53.8)
Q10: The app adequately acknowledged and provided information to let me know the progress of my action.	1 (0.5)	1 (0.5)	2 (0.9)	17 (8.1)	14 (6.7)	70 (33.3)	105 (50.0)
Q11: The navigation was consistent when moving between screens.	3 (1.4)	1 (0.5)	4 (1.9)	22 (10.5)	20 (9.5)	67 (31.9)	93 (44.3)
Q12: The interface of the app allowed me to use all the functions (such as entering information, responding to reminders, viewing information) offered by the app.	2 (0.9)	3 (1.4)	6 (2.9)	24 (11.4)	22 (10.5)	63 (30.0)	90 (42.9)
Q13: This app has all the functions and capabilities I expect it to have.	2 (0.9)	1 (0.5)	10 (4.8)	20 (9.5)	20 (9.5)	74 (35.2)	83 (39.5)
Q14: Whenever I made a mistake using the app, I could recover easily and quickly.	5 (2.4)	4 (1.9)	6 (2.9)	41 (19.5)	17 (8.1)	60 (28.6)	77 (36.7)
Q15: The app would be useful for my health and well-being.	1 (0.5)	2 (0.9)	1 (0.5)	9 (4.3)	10 (4.8)	71 (33.8)	116 (55.2)
Q16: The app improved my access to health care services.	1 (0.5)	2 (0.9)	1 (0.5)	12 (5.7)	13 (6.2)	78 (37.1)	103 (49.1)
Q17: The app helped me manage my health effectively.	1 (0.5)	2 (0.9)	1 (0.5)	15 (7.1)	16 (7.6)	77 (36.7)	98 (46.7)
Q18: The app made it convenient for me to communicate with my health care provider.	1 (0.5)	2 (0.9)	1 (0.5)	13 (6.2)	7 (3.3)	67 (31.9)	119 (56.7)
Q19: Using the app, I had many more opportunities to interact with my health care provider.	2 (0.9)	1 (0.5)	2 (0.9)	18 (8.6)	15 (7.1)	65 (31.0)	107 (51.0)
Q20: I felt confident that any information I sent to my provider using the app would be received.	1 (0.5)	2 (0.9)	1 (0.5)	15 (7.1)	11 (5.2)	64 (30.5)	116 (55.2)
Q21: I felt comfortable communicating with my health care provider using the app.	1 (0.5)	2 (0.9)	1 (0.5)	15 (7.1)	8 (3.8)	59 (28.1)	124 (59.1)

### Exploratory Factor Analysis

The construct validity of the Mx-MAUQ was assessed using a polychoric correlation matrix, which revealed moderate to very high positive correlations among all items, with correlation coefficients ranging from 0.38 to 0.91 (see Multimedia Appendix 3). The appropriateness of the factor analysis was corroborated by a high sampling adequacy measure (KMO=0.94) and a significant result from Bartlett’s test of sphericity (*P*<.001).

Table 3 presents the results of the factor analysis, including item loadings for the rotated factor solution, items’ communality, and the percentage of total variance explained. The analysis identified 2 factors, with all items retained in the final version of the Mx-MAUQ, as they had acceptable loadings (greater than 0.40) and did not show association with more than 1 factor. The communalities for all 21 items ranged from 0.62 to 0.91. The 2 identified factors together explained 91.6% of the variance.

**Table 3.** Factor analysis and internal consistency of the Mexican version of the interactive mHealth App Usability Questionnaire (Mx-MAUQ) (N=210).

Items	Factor loadings		Communality
	Ease of use	Satisfaction, usefulness, and system information arrangement	
Q1: It was easy for me to learn to use the app.	0.93	−0.18	0.67
Q2: I like the interface of the app.	0.53	0.39	0.71
Q3: The app was easy to use.	0.94	−0.12	0.75
Q4: The information in the app was well organized, so I could easily find the information I needed.	0.66	0.25	0.72
Q5: I feel comfortable using this app in social settings.	0.69	0.28	0.80
Q6: The amount of time involved in using this app has been fitting for me.	0.72	0.20	0.75
Q7: I would use this app again.	0.29	0.67	0.79
Q8: Overall, I am satisfied with this app.	0.31	0.70	0.87
Q9: This mHealth app provided an acceptable way to receive health care services.	0.05	0.90	0.88
Q10: The app adequately acknowledged and provided information to let me know the progress of my action.	0.12	0.81	0.80
Q11: The navigation was consistent when moving between screens.	0.73	0.23	0.81
Q12: The interface of the app allowed me to use all the functions (such as entering information, responding to reminders, viewing information) offered by the app.	0.67	0.30	0.81
Q13: This app has all the functions and capabilities I expect it to have.	0.29	0.59	0.65
Q14: Whenever I made a mistake using the app, I could recover easily and quickly.	0.67	0.16	0.62
Q15: The app would be useful for my health and well-being.	0.12	0.85	0.87
Q16: The app improved my access to health care services.	0.11	0.82	0.80
Q17: The app helped me manage my health effectively.	0.00	0.95	0.91
Q18: The app made it convenient for me to communicate with my health care provider.	−0.01	0.92	0.85
Q19: Using the app, I had many more opportunities to interact with my health care provider.	−0.02	0.93	0.84
Q20: I felt confident that any information I sent to my provider using the app would be received.	−0.03	0.88	0.74
Q21: I felt comfortable communicating with my health care provider using the app.	0.01	0.85	0.73
			Total
% of the total variance	9.1	82.5	91.6
Cronbach α	.94	.97	.97

### Internal Consistency

The first factor, “Ease of Use,” consists of 9 items (1-6, 11, 12, and 14) and has a Cronbach α of .94. The second factor, “Satisfaction, Usefulness, and System Information Arrangement,” includes 12 items (7-10, 13, and 15-21) and has a Cronbach α of .97. The overall Cronbach α for all items in the Mx-MAUQ is 0.97. The presence of only 2 factors in MX-MAUQ is different from the original MAUQ, which has 3 factors, including “Ease of Use and Satisfaction” (items 1‐8), “System Information Arrangement” (items 9‐14) and “Usefulness” (items 15‐21), according to the order of the adapted Mx-MAUQ (as shown in Table 3).

### Discriminant Validity

Table 4 presents the results of the analysis examining the differentiation of Mx-MAUQ scores across subgroups. The findings indicate that women with an education level of high school or higher reported significantly higher scores for the 2 factors, as well as a higher total Mx-MAUQ score, compared to women with less than a secondary education. No statistically significant differences were observed among the other subgroups.

**Table 4.** Differentiation of the Mexican version of the interactive mHealth App Usability Questionnaire (Mx-MAUQ) (N=210).

Variable	Ease of use			Satisfaction, usefulness, and system information arrangement			Total score		
	Median	Z statistic^a^	*P* value	Median	Z statistic^a^	*P* value	Median	Z statistic^a^	*P* value
Age group									
<60 years	6.2	1.71	.09	6.5	−0.38	.70	6.4	0.90	.36
≥60 years	6.0			6.5			6.0		
Educational attainment									
Secondary school or more	5.9	−2.60	.009	6.2	−2.35	.02	6.0	−2.71	.006
High school or more	6.2			6.7			6.5		
Chronic comorbidity									
No	6.2	1.52	.13	6.5	−0.08	.93	6.4	0.60	.54
Yes	6.0			6.5			6.0		
Cancer stage									
I-II	6.1	0.06	.95	6.7	1.23	.22	6.3	0.36	.72
III	6.1			6.4			6.2		
Treatment type									
Radiation therapy	6.3	1.16	.25	6.4	−0.86	.39	6.3	0.26	.79
Chemotherapy	6.1			6.6			6.3		

a Wilcoxon rank- sum test

## Discussion

### Principal Findings

The MX-MAUQ, adapted and validated for a web application for women with breast cancer in Mexico, demonstrated acceptable psychometric characteristics. The MX-MAUQ content validity was confirmed by an expert panel and cognitive interviews with women, who suggested minor changes for clarity. The EFA identified 2 factors explaining 91.6% of the variance and retaining all items. The “Ease of Use” factor had 9 items, and “Satisfaction, Usefulness, and System Information Arrangement” contained 12 items. The reliability showed a Cronbach α above .9 for each factor and the overall questionnaire. Women with higher education levels scored significantly higher on the Mx-MAUQ than those with lower educational attainment.

The results showed a strong relevance of Mx-MAUQ content for evaluation of responsive web applications. The content validity results in our research (CVI from 0.75 to 1.00) were consistent with the study by Zhao et al [17] in China, where an item-level CVI varied from 0.85 to 1.00. Experts have found that the MAUQ questions are general enough to be relevant not only to mobile apps or specific medical conditions but also to a variety of digital platforms and health issues. While we did not use a face validity index to assess how understandable the questionnaire was for users, as was done in the study by Mustafa et al [19] in Malaysia, the results from cognitive interviews confirmed understandability of most items and resulted in minor adjustments to the final Mx-MAUQ. These findings are consistent with previous validation of the MAUQ to assess multiple apps designed for different purposes and user groups, like the “Left-handed Doctor” mHealth app in China for medical consultations and orientation of the general population [18], LactApp for women in Spain to address their breastfeeding-related concerns [16], the Enable app for cancer patients in Germany for ePRO registration and monitoring the side effects of treatment [14], the DIGICOG-MS app for cognitive evaluation of individuals with multiple sclerosis in Italy [15], and the MyFitnessPal app in Malaysia to determine the caloric intake and weight goals and provide nutritional advice for the app users [19]. In light of this evidence, the MAUQ and Mx-MAUQ could potentially be applied to any mHealth or eHealth app designed for different populations.

The EFA confirmed the retention of all 21 items. This finding aligns with previous studies where no items were discarded [17,18]. However, the items were grouped into just 2 factors (domains): “Ease of Use” and “Satisfaction, Usefulness, and System Information Arrangement,” which is different from the 3 factors identified in the American MAUQ and a Chinese adaptation [13,17]. In the current research, the domain “System Information Arrangement” was not maintained as an individual factor; its components were incorporated into the “Ease of Use” domain (the first 6 items) and “Satisfaction, Usefulness, and System Information Arrangement” Mx-MAUQ domain (the rest of the items). Additionally, questions related to satisfaction left the original “Ease of Use and Satisfaction” domain and passed to the new “Satisfaction, Usefulness, and System Information Arrangement” domain. These differences could be attributed to characteristics of our sample, which included only women with breast cancer. Previous studies identified that limiting data heterogeneity can reduce the numbers of factors retained [30].

The analysis of the polychoric correlation matrix showed a positive correlation among all items, with values ranging from 0.38 to 0.91. This result aligns with the study conducted by Shan et al [18], which also reported significant positive correlations among the 21 items, ranging from 0.68 to 0.92. Additionally, similar to the studies conducted in China [17,18], our study showed a KMO measure above 0.9, indicating a good sample adequacy for EFA. Furthermore, we used a cutoff value of 0.4 to determine the factor loadings for the items, and no items displayed multiple factor loadings exceeding this threshold. This contrasts with the original version validated by Zhou et al [13], which used a lower cutoff point of 0.32. In their study, some items had factor loadings lower than 0.32 or multiple factor loadings greater than the cutoff value; however, the authors chose to retain those items based on their previous experience in mHealth app usability studies. The Mx-MAUQ accounted for 91.6% of the variability in the items, a variance value that is significantly higher than the 63.8% reported in the validation study by Zhao et al [17] for the 21-item scale.

We identified strong internal consistency of the Mx-MAUQ with an overall Cronbach α of .97. The values for Domain 1 and Domain 2 were 0.97 and 0.94, respectively. These results are consistent with the original version of the MAUQ, which had a Cronbach α of .93, and with other adaptations of the same questionnaire [13,17,18].

The Mx-MAUQ demonstrated superior psychometric characteristics compared to previously adapted and validated usability tools in Mexico. For example, Sevilla-González et al [10] validated the SUS for adults using a dietary website and focused solely on the content validity and reliability of the questionnaire, reporting a Cronbach α of .81. Hedlefs-Aguilar et al [11,12] evaluated both the SUS and CSUQ among university students using an educational platform. The researchers found that EFA indicated the SUS accounted for only 52.8% of the explained variance, with a Cronbach α of .59, while the CSUQ explained 77.5% of the variance and had a Cronbach α of .96. In contrast, our study achieved significantly better results, with EFA showing 91.6% of explained variance and a Cronbach α of .97.

Most women rated the breast cancer application very positively, achieving higher usability scores than those found in an MAUQ validation study in China [18], where users of the “Left-handed Doctor App” mostly answered “agree” to usability items, while our study respondents primarily said, “strongly agree.” This difference likely reflects the more reserved and pragmatic nature of Chinese culture compared to Mexican culture, resulting in more favorable evaluations of the “OncoMama App” [31]. Given the substantial correlation among the existing items and the consistently high scores observed, future research is recommended to test a short form of this questionnaire by eliminating items that convey similar meanings.

Additionally, our analysis of Mx-MAUQ results by subgroups revealed that women with low levels of education experienced lower usability with mHealth apps compared to those with higher educational attainment. These differences may be due to the challenges that individuals with less schooling face when using digital tools, such as navigational difficulties and those related to interpreting and analyzing information [32]. To ensure these tools are used effectively, digital literacy training is necessary for those with low levels of schooling.

We expect that using the adopted and validated Mx-MAUQ to assess responsive web applications will enable a comprehensive evaluation of usability, usefulness, satisfaction, and the organization of system information for emerging eHealth apps in Mexico. This will assist digital health developers and researchers in refining their eHealth innovations by identifying and addressing the challenges faced by users. Additionally, policymakers can leverage insights from this questionnaire to select and integrate innovative, user-friendly apps into health care practice, thereby enhancing the quality of care for both patients and providers.

**Limitations**

The study has several limitations. First, the validation of the Mx-MAUQ was conducted exclusively with women diagnosed with breast cancer at 4 IMSS hospitals in 2 Mexican cities. This could restrict the generalizability of the results to a wider population, potentially underrepresenting the varied socioeconomic backgrounds of breast cancer patients throughout Mexico, as IMSS covers primarily those from the formal labor market and their families, excluding women from the informal labor sector, particularly those living in rural areas who may lack reliable internet access. We also excluded illiterate women or those with cognitive disabilities. We believe these women would need a specialized application that takes into account their illiteracy and disability and is likely to center on their caregivers to record supportive care needs and negative effects of treatment and provide care according to the application and nurses’ recommendations or use the voice function to register symptoms and to provide recommendations. Therefore, future studies could benefit from Mx-MAUQ validation in caregivers of illiterate patients and those with visual or mental disabilities to reach broader generalizability of the results.

Second, unlike the American and Italian versions of the questionnaire [13,15], this study did not examine the convergent validity of the Mx-MAUQ in relation to other similar instruments that assess usability, which is an important aspect of construct validation [33]. Third, we did not conduct confirmatory factor analysis (CFA) to validate the factor structure with an additional independent sample because our pilot study and clinical trial of the OncoMama app included only 50 and 205 patients, leaving us short of the other 210 needed for CFA. Future studies should confirm the Mx-MAUQ’s 2-domain structure using CFA with a larger, more diverse Mexican sample that includes both urban and rural users, as well as caregivers of illiterate and disabled patients. Finally, we did not assess usability over an extended period, so future research could include longitudinal evaluations to track changes in usability perceptions.

**Strengths**

The study’s strengths include the absence of incomplete data and the inclusion of a diverse sample of breast cancer patients regarding age, educational background, chronic comorbidities, cancer stage, and treatment type. Additionally, we conducted subgroup analyses that enhanced the validity of the Mx-MAUQ assessment.

### Conclusions

Mx-MAUQ showed satisfactory psychometric properties based on EFA, internal consistency, and discriminant analysis, making it a suitable tool for a comprehensive assessment of the usability of interactive web-based eHealth applications for women with breast cancer in Mexico. Additionally, this research can be a benchmark for the validation of the interactive version of MAUQ in other Spanish-speaking countries, with the goal of evaluating the usability of eHealth web and mHealth apps for patients with cancer or individuals with other chronic diseases.

## Supplementary material

10.2196/72215 Multimedia Appendix 1 Mexican version of the interactive version of the mHealth App Usability Questionnaire (Mx-MAUQ). Mexican Spanish translation.

10.2196/72215 Multimedia Appendix 2 Content validity of the Mexican version of the interactive version of the mHealth App Usability Questionnaire (Mx-MAUQ).

10.2196/72215 Multimedia Appendix 3 Polychoric correlation matrix of the Mexican version of the interactive version of the mHealth App Usability Questionnaire (Mx-MAUQ).

## Abbreviations

CFA: confirmatory factor analysis
CSUQ: Computer Systems Usability Questionnaire
CVI: content validity index
EFA: exploratory factor analysis
IMSS: Mexican Institute of Social Security
KMO: Kaiser-Meyer-Olkin
MAUQ: mHealth App Usability Questionnaire
mHealth: mobile health
Mx-MAUQ: Mexican version of the interactive mHealth App Usability Questionnaire
SUS: System Usability Scale

## References

[1] (2019). WHO guideline recommendations on digital interventions for health system strengthening.

[2] (2018). Digital health resolution. Seventy-first World Health Assembly.

[3] Lee JY, Kim JY, You SJ (2019). Development and usability of a life-logging behavior monitoring application for obese patients. J Obes Metab Syndr.

[4] (2022). Cancer today. International Agency for Research on Cancer (IARC).

[5] Doubova SV, Aguirre-Hernandez R, Gutiérrez-de la Barrera M, Infante-Castañeda C, Pérez-Cuevas R (2015). Supportive care needs of Mexican adult cancer patients: validation of the Mexican version of the Short-Form Supportive Care Needs Questionnaire (SCNS-SFM). Support Care Cancer.

[6] Doubova SV, Martinez-Vega IP, Infante-Castañeda C, Aranda-Flores CE, Knaul FM, Pérez-Cuevas R (2021). Social inequalities in supportive care needs and quality of patient-centered care of cancer patients in Mexico. Support Care Cancer.

[7] Contreras Sánchez SE, Doubova SV, Grajales Álvarez R (2023). Design and evaluation of a digital health intervention with proactive follow-up by nurses to improve healthcare and outcomes for patients with breast cancer in Mexico: protocol for a randomised clinical trial. BMJ Open.

[8] Turner-McGrievy GM, Hales SB, Schoffman DE (2017). Choosing between responsive-design websites versus mobile apps for your mobile behavioral intervention: presenting four case studies. Transl Behav Med.

[9] Contreras Sánchez SE, Doubova SV, Martinez Vega IP (2024). Addressing the unmet needs of women with breast cancer in Mexico: a non-randomised pilot study of the digital ePRO intervention. BMJ Open.

[10] Sevilla-Gonzalez MDR, Moreno Loaeza L, Lazaro-Carrera LS (2020). Spanish version of the System Usability Scale for the assessment of electronic tools: development and validation. JMIR Hum Factors.

[11] Hedlefs Aguilar MI, Garza Villegas AA (2016). Análisis comparativo de la Escala de Usabilidad del Sistema (EUS) en dos versiones [Article in Spanish]. RECI.

[12] Hedlefs Aguilar MI, De la Garza González A, Sánchez Miranda MP, Garza Villegas AA (2015). Adaptación al español del Cuestionario de Usabilidad de Sistemas Informáticos CSUQ [Article in Spanish]. RECI.

[13] Zhou L, Bao J, Setiawan IMA, Saptono A, Parmanto B (2019). The mHealth App Usability Questionnaire (MAUQ): development and validation study. JMIR Mhealth Uhealth.

[14] Moorthy P, Weinert L, Harms BC, Anders C, Siegel F (2023). German version of the mHealth App Usability Questionnaire in a cohort of patients with cancer: translation and validation study. JMIR Hum Factors.

[15] Podda J, Grange E, Susini A (2024). Italian version of the mHealth App Usability Questionnaire (Ita-MAUQ): translation and validation study in people with multiple sclerosis. JMIR Hum Factors.

[16] Quifer-Rada P, Aguilar-Camprubí L, Gómez-Sebastià I, Padró-Arocas A, Mena-Tudela D (2023). Spanish version of the mHealth app usability questionnaire (MAUQ) and adaptation to breastfeeding support apps. Int J Med Inform.

[17] Zhao S, Cao Y, Cao H (2022). Chinese version of the mHealth App Usability Questionnaire: cross-cultural adaptation and validation. Front Psychol.

[18] Shan Y, Ji M, Xie W (2022). Chinese version of the Mobile Health App Usability Questionnaire: translation, adaptation, and validation study. JMIR Form Res.

[19] Mustafa N, Safii NS, Jaffar A (2021). Malay version of the mHealth App Usability Questionnaire (M-MAUQ): translation, adaptation, and validation study. JMIR Mhealth Uhealth.

[20] WHODAS 2.0 translation package (version 1.0) translation and linguistic evaluation protocol and supporting material. World Health Organization.

[21] Lynn MR (1986). Determination and quantification of content validity. Nurs Res.

[22] Almanasreh E, Moles R, Chen TF (2019). Evaluation of methods used for estimating content validity. Res Social Adm Pharm.

[23] Scott K, Ummer O, LeFevre AE (2021). The devil is in the detail: reflections on the value and application of cognitive interviewing to strengthen quantitative surveys in global health. Health Policy Plan.

[24] Willis G, Johnson TP (2014). Handbook of Health Survey Methods United States of America.

[25] Worthington R, Whittaker T (2006). Scale development research: a content analysis and recommendations for best practices. Couns Psychol.

[26] Mislevy RJ (1986). Recent developments in the factor analysis of categorical variables. J Educ Stat.

[27] Pizarro K, Martínez O (2020). Análisis factorial exploratorio mediante el uso de las medidas de adecuación muestral KMO y esfericidad de Bartlett para determinar factores principales. J Sci Res.

[28] Hair JF, Anderson RE, Tatham RL, Black WC (1999). Análisis Multivariante.

[29] Jaju A, Crask MR, Menon A, Sharma A (1999). AMA Winter Educators’ Conference: Marketing Theory & Application.

[30] Gaskin CJ, Lambert SD, Bowe SJ, Orellana L (2017). Why sample selection matters in exploratory factor analysis: implications for the 12-item World Health Organization Disability Assessment Schedule 2.0. BMC Med Res Methodol.

[31] Cervantes Zepeda M, Lemus Delgado D (2015). China y México: Desempeño económico y valores éticos religiosos. Méx Cuenca Pac.

[32] Guimarães L, Martins N, Brandão D (2021). Interface design for low schooling and low literacy users: the ECHO project. Convergências.

[33] Boateng GO, Neilands TB, Frongillo EA, Melgar-Quiñonez HR, Young SL (2018). Best practices for developing and validating scales for health, social, and behavioral research: a primer. Front Public Health.

[34] GitHub, Inc.

