# Supplementary material for: The Mexican Version of the Interactive mHealth App Usability Questionnaire (Mx-MAUQ) in Women With Breast Cancer: Instrument Validation Study
Source: J Med Internet Res. 2025 Aug 29;27:e72215. doi: 10.2196/72215 (PMC12396799; doi:10.2196/72215)
Supplement: Multimedia Appendix 1 [file jmir-v27-e72215-s001.docx]

**Multimedia Appendix 1. Mexican version of the interactive version of the mHealth App Usability Questionnaire (Mx-MAUQ). Mexican Spanish translation.**

| N° | Item | Totalmente en desacuerdo | En  Desacuerdo | Algo en desacuerdo | Neutral:  ni acuerdo ni desacuerdo | Algo de acuerdo | De  Acuerdo | Totalmente  de acuerdo |
| --- | --- | --- | --- | --- | --- | --- | --- | --- |
| 1 | Fue fácil para mí aprender a usar la aplicación. | 1 | 2 | 3 | 4 | 5 | 6 | 7 |
| 2 | Me gusta la interfaz de la aplicación (forma visual en la que se presentan los contenidos). | 1 | 2 | 3 | 4 | 5 | 6 | 7 |
| 3 | La aplicación fue fácil de usar. | 1 | 2 | 3 | 4 | 5 | 6 | 7 |
| 4 | La información de la aplicación estaba bien organizada, por lo que pude encontrar fácilmente la información que necesitaba. | 1 | 2 | 3 | 4 | 5 | 6 | 7 |
| 5 | Me siento cómoda usando esta aplicación en diferentes entornos (ambientes) sociales. | 1 | 2 | 3 | 4 | 5 | 6 | 7 |
| 6 | La cantidad de tiempo que tengo que usar esta aplicación ha sido adecuada para mí. | 1 | 2 | 3 | 4 | 5 | 6 | 7 |
| 7 | Volvería a usar esta aplicación. | 1 | 2 | 3 | 4 | 5 | 6 | 7 |
| 8 | En general, estoy satisfecha con esta aplicación. | 1 | 2 | 3 | 4 | 5 | 6 | 7 |
| 9 | Esta aplicación digital me proporcionó una forma útil para recibir atención de los servicios de salud. | 1 | 2 | 3 | 4 | 5 | 6 | 7 |
| 10 | La aplicación digital identifica adecuadamente y proporciona la información para recomendarme las actividades que tengo que hacer para sentirme mejor. | 1 | 2 | 3 | 4 | 5 | 6 | 7 |
| 11 | La navegación entre pantallas de la aplicación es fácil. | 1 | 2 | 3 | 4 | 5 | 6 | 7 |
| 12 | La aplicación me permitió usar todas las funciones que ofrece (como ingresar información, responder recordatorios, ver información). | 1 | 2 | 3 | 4 | 5 | 6 | 7 |
| 13 | Esta aplicación tiene todas las funciones y capacidades que espero que tenga. | 1 | 2 | 3 | 4 | 5 | 6 | 7 |
| 14 | Siempre que cometía un error al usar la aplicación (error en registro de los datos), pude identificarlo y corregirlo fácil y rápidamente. | 1 | 2 | 3 | 4 | 5 | 6 | 7 |
| 15 | La aplicación sería útil para mi salud y bienestar. | 1 | 2 | 3 | 4 | 5 | 6 | 7 |
| 16 | La aplicación mejoró mi acceso a la atención de los servicios de salud. | 1 | 2 | 3 | 4 | 5 | 6 | 7 |
| 17 | La aplicación me ayudó a cuidar mi salud de manera efectiva. | 1 | 2 | 3 | 4 | 5 | 6 | 7 |
| 18 | La aplicación me ayudó a comunicarme con el personal de salud responsable. | 1 | 2 | 3 | 4 | 5 | 6 | 7 |
| 19 | Al usar la aplicación, tuve muchas más oportunidades de interactuar con los profesionales de salud. | 1 | 2 | 3 | 4 | 5 | 6 | 7 |
| 20 | Estaba segura de que recibiría respuesta a la información que le enviara al personal de salud mediante la aplicación. | 1 | 2 | 3 | 4 | 5 | 6 | 7 |
| 21 | Me sentí cómoda comunicándome con el personal de salud mediante la aplicación. | 1 | 2 | 3 | 4 | 5 | 6 | 7 |
