# Supplementary material for: The Mexican Version of the Interactive mHealth App Usability Questionnaire (Mx-MAUQ) in Women With Breast Cancer: Instrument Validation Study
Source: J Med Internet Res. 2025 Aug 29;27:e72215. doi: 10.2196/72215 (PMC12396799; doi:10.2196/72215)
Supplement: Multimedia Appendix 2 [file jmir-v27-e72215-s002.docx]

**Multimedia Appendix 2. Content validity of the Mexican version of the interactive version of the mHealth App Usability Questionnaire (Mx-MAUQ).**

| Item | Number of experts who deemed the item relevant (ne) / Total number of experts (N) | Content validity index (CVI)  (ne - N/2) / (N/2) |
| --- | --- | --- |
| Q1 | 8 / 8 | 1.00 |
| Q2 | 8 / 8 | 1.00 |
| Q3 | 8 / 8 | 1.00 |
| Q4 | 8 / 8 | 1.00 |
| Q5 | 7 / 8 | 0.75 |
| Q6 | 8 / 8 | 1.00 |
| Q7 | 8 / 8 | 1.00 |
| Q8 | 8 / 8 | 1.00 |
| Q9 | 7 / 8 | 0.75 |
| Q10 | 8 / 8 | 1.00 |
| Q11 | 8 / 8 | 1.00 |
| Q12 | 8 / 8 | 1.00 |
| Q13 | 8 / 8 | 1.00 |
| Q14 | 7 / 8 | 0.75 |
| Q15 | 8 / 8 | 1.00 |
| Q16 | 8 / 8 | 1.00 |
| Q17 | 8 / 8 | 1.00 |
| Q18 | 8 / 8 | 1.00 |
| Q19 | 8 / 8 | 1.00 |
| Q20 | 8 / 8 | 1.00 |
| Q21 | 8 / 8 | 1.00 |
