# Supplementary material for: The Mexican Version of the Interactive mHealth App Usability Questionnaire (Mx-MAUQ) in Women With Breast Cancer: Instrument Validation Study
Source: J Med Internet Res. 2025 Aug 29;27:e72215. doi: 10.2196/72215 (PMC12396799; doi:10.2196/72215)
Supplement: Multimedia Appendix 3 [file jmir-v27-e72215-s003.docx]

**Multimedia Appendix 3. Polychoric correlation matrix of the Mexican version of the interactive version of the mHealth App Usability Questionnaire (Mx-MAUQ).**

| Items | Item 01 | Item 02 | Item 03 | Item 04 | Item 05 | Item 06 | Item 07 | Item 08 | Item 09 | Item 10 | Item 11 |
| --- | --- | --- | --- | --- | --- | --- | --- | --- | --- | --- | --- |
| Item 01 | 1.00 |  |  |  |  |  |  |  |  |  |  |
| Item 02 | 0.57 | 1.00 |  |  |  |  |  |  |  |  |  |
| Item 03 | 0.83 | 0.68 | 1.00 |  |  |  |  |  |  |  |  |
| Item 04 | 0.60 | 0.75 | 0.69 | 1.00 |  |  |  |  |  |  |  |
| Item 05 | 0.64 | 0.73 | 0.66 | 0.78 | 1.00 |  |  |  |  |  |  |
| Item 06 | 0.62 | 0.70 | 0.61 | 0.76 | 0.87 | 1.00 |  |  |  |  |  |
| Item 07 | 0.50 | 0.69 | 0.51 | 0.68 | 0.78 | 0.74 | 1.00 |  |  |  |  |
| Item 08 | 0.54 | 0.76 | 0.56 | 0.73 | 0.81 | 0.76 | 0.91 | 1.00 |  |  |  |
| Item 09 | 0.44 | 0.70 | 0.54 | 0.66 | 0.71 | 0.65 | 0.81 | 0.84 | 1.00 |  |  |
| Item 10 | 0.39 | 0.72 | 0.52 | 0.63 | 0.73 | 0.67 | 0.79 | 0.84 | 0.90 | 1.00 |  |
| Item 11 | 0.66 | 0.77 | 0.78 | 0.75 | 0.78 | 0.76 | 0.74 | 0.78 | 0.71 | 0.68 | 1.00 |
| Item 12 | 0.61 | 0.76 | 0.69 | 0.77 | 0.82 | 0.79 | 0.72 | 0.78 | 0.72 | 0.74 | 0.82 |
| Item 13 | 0.43 | 0.68 | 0.52 | 0.64 | 0.67 | 0.64 | 0.73 | 0.78 | 0.75 | 0.72 | 0.70 |
| Item 14 | 0.60 | 0.58 | 0.62 | 0.64 | 0.70 | 0.71 | 0.64 | 0.62 | 0.54 | 0.56 | 0.71 |
| Item 15 | 0.46 | 0.77 | 0.51 | 0.69 | 0.71 | 0.68 | 0.82 | 0.91 | 0.85 | 0.83 | 0.71 |
| Item 16 | 0.46 | 0.73 | 0.54 | 0.61 | 0.65 | 0.61 | 0.79 | 0.80 | 0.83 | 0.79 | 0.68 |
| Item 17 | 0.42 | 0.74 | 0.49 | 0.63 | 0.68 | 0.62 | 0.82 | 0.85 | 0.90 | 0.87 | 0.68 |
| Item 18 | 0.43 | 0.65 | 0.52 | 0.65 | 0.63 | 0.58 | 0.73 | 0.79 | 0.87 | 0.81 | 0.66 |
| Item 19 | 0.46 | 0.67 | 0.50 | 0.64 | 0.64 | 0.59 | 0.68 | 0.75 | 0.85 | 0.78 | 0.62 |
| Item 20 | 0.38 | 0.58 | 0.42 | 0.57 | 0.64 | 0.57 | 0.74 | 0.78 | 0.78 | 0.71 | 0.60 |
| Item 21 | 0.47 | 0.60 | 0.48 | 0.63 | 0.61 | 0.58 | 0.70 | 0.73 | 0.79 | 0.67 | 0.61 |
|  | Item 12 | Item 13 | Item 14 | Item 15 | Item 16 | Item 17 | Item 18 | Item 19 | Item 20 | Item 21 |  |
| Item 12 | 1.00 |  |  |  |  |  |  |  |  |  |  |
| Item 13 | 0.69 | 1.00 |  |  |  |  |  |  |  |  |  |
| Item 14 | 0.74 | 0.60 | 1.00 |  |  |  |  |  |  |  |  |
| Item 15 | 0.72 | 0.75 | 0.59 | 1.00 |  |  |  |  |  |  |  |
| Item 16 | 0.70 | 0.76 | 0.63 | 0.85 | 1.00 |  |  |  |  |  |  |
| Item 17 | 0.67 | 0.78 | 0.60 | 0.89 | 0.86 | 1.00 |  |  |  |  |  |
| Item 18 | 0.70 | 0.67 | 0.57 | 0.83 | 0.82 | 0.86 | 1.00 |  |  |  |  |
| Item 19 | 0.66 | 0.67 | 0.55 | 0.81 | 0.80 | 0.87 | 0.91 | 1.00 |  |  |  |
| Item 20 | 0.65 | 0.64 | 0.54 | 0.75 | 0.78 | 0.79 | 0.80 | 0.80 | 1.00 |  |  |
| Item 21 | 0.59 | 0.55 | 0.52 | 0.78 | 0.71 | 0.77 | 0.85 | 0.88 | 0.81 | 1.00 |  |
